# Bioactivity Profiling and Phytochemical Analysis of *Carissa carandas* Extracts: Antioxidant, Anti-Inflammatory, and Anti-Urinary Tract Infection Properties

**DOI:** 10.3390/antiox13091037

**Published:** 2024-08-27

**Authors:** Wisha Saeed, Tariq Ismail, Muhammad Qamar, Tuba Esatbeyoglu

**Affiliations:** 1 Department of Food Science and Technology, Faculty of Food Science and Nutrition, Bahauddin Zakariya University, Multan 60800, Pakistan; wishasaeed1@gmail.com (W.S.); tariqismail@bzu.edu.pk (T.I.); 2 Department of Molecular Food Chemistry and Food Development, Institute of Food and One Health, Gottfried Wilhelm Leibniz University Hannover, Am Kleinen Felde 30, 30167 Hannover, Germany

**Keywords:** phenolic acids, anthocyanins, oxidation, inflammation, urinary tract infections, bioassay-guided technique, LC-MS, HPLC

## Abstract

*Carissa carandas* L. (Apocynaceae) is widely distributed in tropical and subtropical regions of Asia including Pakistan, India, Afghanistan, and Sri Lanka. *C. carandas* is considered as an integral component of traditional medicinal systems to combat several health ailments. The present study aimed to assess this plant’s phytochemical contents and biological potential by performing sequential extraction, adopting a bioassay-guided approach. *C. carandas* powder was extracted with *n*-hexane to remove fatty substances and then residues were sequentially extracted with dichloromethane, methanol, and 50% methanol. All the sequential crude extracts were evaluated for phytochemical contents (total phenolics, flavonoids, and anthocyanins), in vitro antioxidant activity (FRAP, DPPH), in vitro anti-inflammatory activity (serum and egg albumin denaturation), in vivo anti-inflammatory activity (carrageenan- and formaldehyde-induced paw edema), and in vitro antimicrobial activity. Active crude extract was then partitioned using the liquid-liquid separation method followed by further separation of the active fraction by RP-HPLC. The active fraction was then subjected to LC-ESI-MS/MS analysis for tentative identification of bioactive metabolites responsible for its bioactive properties, followed by HPLC quantification. The analysis revealed methanol extract to have more phytochemical contents, radical scavenging properties, reduced inflammation in both models (in vitro and in vivo), and antimicrobial properties against urinary tract infection-causing agents as compared to dichloromethane and 50% methanol extracts. The ethyl acetate fraction obtained after liquid-liquid partitioning (LLP) of the active methanol extract exhibited more activity as compared to *C. carandas* methanol extract. RP-HPLC sub-fractionation yielded seven sub-fractions, but a slight decrease in biological potential was recorded. Therefore, LLP fraction B was subjected to further analysis. LC-ESI-MS/MS analysis led to the tentative identification of phenolic acids (chlorogenic acid, quinic acid), flavonoids (quercetin), and anthocyanins (peonidin-3-arabinoside, delphinidin-3-galactoside, delphinidin-3-rutinoside) in the active LLP ethyl acetate fraction. Chlorogenic acid, ellagic acid, and quinic acid were quantified as 17.6 µg/mg, 5.90 µg/mg, and 3.30 µg/mg, respectively, on a dry weight basis by HPLC. *C. carandas* may be considered a promising therapeutic plant, and the results of the current study provide more evidence to support the assertions made in ancient medical traditions. These findings highlight its promising applications in health, medicine, cosmetics, preservatives, and as a natural coloring agent.

## 1. Introduction

Oxidative stress, which affects lipids, proteins, and DNA, is linked to cardiovascular, inflammatory, and neurodegenerative ailments, and the process of ageing [1,2]. Synthetic antioxidants, including butylated hydroxyanisole and butylated hydroxy-toluene are taken in food, medicines, and cosmetics to avoid oxidative reactions, as well as to treat maladies associated with oxidative stress. Nevertheless, there is increasing apprehension over their safety due to several studies that have demonstrated mutagenic and carcinogenic properties connected to some synthetic antioxidants [3]. Similarly, nonsteroidal drugs (NSAIDS) and corticosteroids are widely used to provide relief from inflammation-related pain, but when taken for longer periods of time can induce gastric problems and disturb the normal functioning of the kidney and liver [4,5]. Additionally, bacterial diseases, mainly urinary tract infections (UTIs), are very common; it is estimated that around 150 million people across the world suffer UTIs every year [6]. For instance, over 11 million people in the United States receive treatments for UTIs annually, with a total cost of USD 6 million. UTIs comprise a significant portion of healthcare-associated infections (HAIs) in Europe, accounting for 19.0% of all HAIs [7,8]. Prevalence of UTIs is greater in women than men, and per reported data, 50% of women and 12% of men develop UTIs in the course of their life [9]. *Escherichia coli*, *Klebsiella pneumoniae*, *Citrobacter*, *Staphylococcus aureus*, *Streptococcus bovis*, *Candida albicans*, and *Candida glabrata* have been established as the main causative agents [10,11]. The treatment includes medicines and hospitalization, resulting in great economic burden. Other than that, frequent use of antibiotics, i.e., antibacterials (imipenem, ciprofloxacin, amikacin, nitrofurantoin) and antifungals (nystatin, fluconazole) can result in antibiotic resistance [12,13]. Presently, multidrug-resistant (MDR) uropathogenic *Escherichia coli* (UPEC) is a major clinical concern, particularly in developing nations. This resistance requires extensive use of broad-spectrum antibiotics, which prolongs hospitalizations and raises treatment expenses. The fast rise of antibiotic resistance in UPEC strains threatens world health care systems [10]. Therefore, non-antibiotic antimicrobial techniques for UTI treatment and prevention are becoming more popular. Alternatives considered are the use of vaccines, bacteriophages, probiotics, and secondary metabolites, the last of which is the main current focus worldwide [14].

*Carissa carandas* (*C. carandas*) belongs to the Apocynaceae family and is an evergreen shrub that remains a less well-known plant with significant economic, commercial, clinical, and nutritional potential. It is also known as “Crane berry” (English), Karonda (Devanagari), Karonda (Hindi), Karamcha (Bengali), and Ci-Huang-Guo (Mandarin Chinese). It is found to be widely distributed in tropical and subtropical regions of Pakistan, India, Sri Lanka, Myanmar, Bangladesh, and Nepal [15]. *C. carandas* berries are widely recognized as a crucial element in ancient medicinal systems for the treatment of various health conditions such as epilepsy, edema, diarrhea, and myopathic spasms [16]. Fruits of *C. carandas* are known to have potential in treating hemorrhoids, appetite loss, calming nervous disorders (as a nervine), colic, splenomegaly, hepatomegaly, amenorrhea, CVDs, and psychiatric anorexia in humans [15]. Its roots are utilized as a bitter stomachic, a vermifuge, and for itching. Traditional medicinal claims regarding *C. carandas* are now substantiated by pharmacological studies that have confirmed its antioxidant [17], anti-inflammatory [18], anticancer [19], antidiabetic [20], and antimicrobial effects [21]. *C. carandas* fruit extracts are reported to contain anthocyanins, phenolic acids, flavonols, alkaloids, and terpenoids [22,23]. These compounds contribute to the plant’s significant biological activity, making it a valuable source. 

However, the aforementioned studies used crude extracts to evaluate the plant’s biological potential. In the present investigation, sequential extraction was performed followed by bioassay-guided fractionation of more potent crude extracts using liquid-liquid partitioning and reverse phase-high performance liquid chromatography to identify the compound or group of compounds responsible for a range of bioactivities including antioxidant, anti-inflammatory, and anti-UTI. Liquid chromatography-mass spectrometry was used to tentatively identify the compounds followed by quantification using external standards by HPLC.

## 2. Material and Methods 

### 2.1. Collection of Berries 

**Figure 1:**
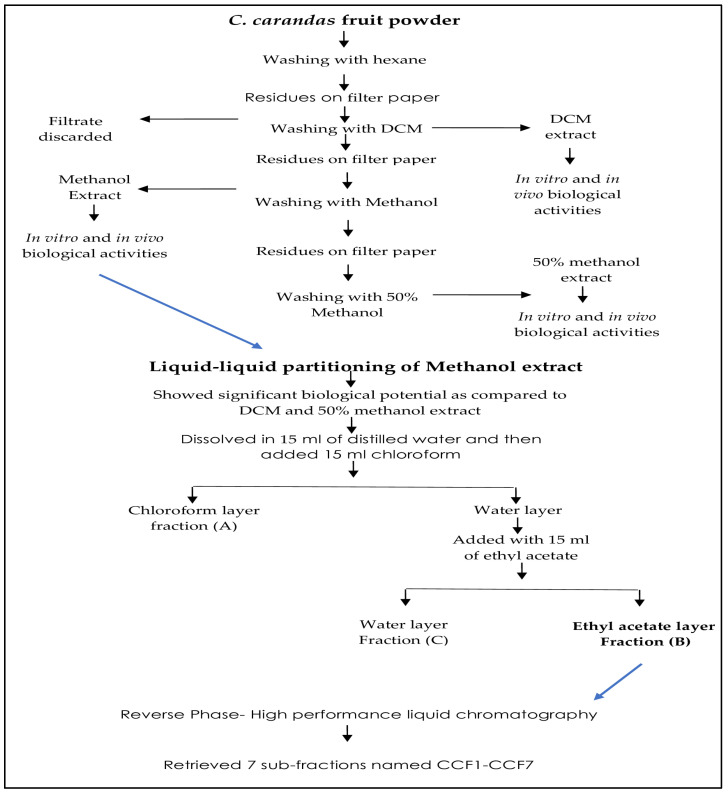
Sequential extraction of *C. carandas*.

The collection *C. Carandas* fruits was done in late July 2023 from the gardens of Bahauddin Zakariya University, Multan, Pakistan, and was subjected to varietal identification. After washing the berries, they were cut into pieces and seeds were removed. *C. carandas* pulp extraction was performed using various solvents on the basis of increasing polarity, i.e., initially with hexane to remove fatty contents, followed by dichloromethane, methanol, and 50% methanol. To obtain the concentrated extracts, the solvents were evaporated in a rotary evaporator (Heidolph, Hei-Vap, Schwabach, Germany). Semi-solid extracts were stored at −70 °C in an ultralow freezer for future experiments. Sequential extraction is well outlined in Figure 1.

### 2.2. Solvents and Reagents 

Standard solvents were employed in the extraction process including gallic acid, quercetin, cyanidin-3-glucoside, indomethacin, and diclofenac sodium, in addition to inflammatory mediators like carrageenan and formaldehyde, all sourced from the local supplier of Sigma Aldrich (Stockholm, Sweden). All chemicals, reagents, and solvents utilized in this study were of analytical grade unless specified otherwise.

### 2.3. Animals

The animal study was performed at the Department of Food Science and Technology, Faculty of Food Science and Nutrition, Multan under the protocol number 02-23 and title “In vivo anti-inflammatory potential of *C. carandas* sequential crude extracts” approved by the Bioethical Committee at Bahauddin Zakariya University for animal usage. Two animals/cage were maintained in a controlled environment (25 °C ± 4 °C, 12/12 light/dark cycles) with ample access to food and water (ad libitum). 

### 2.4. Phytochemicals Characterization 

Total phenolic contents were calculated adopting the Folin Ciocalteu (FC) colorimetric method using gallic acid as standard and ethanol as blank. Briefly, 0.5 mL of test sample was mixed with 1.5 mL Folin–Ciocalteu reagent and 1.2 mL of 7.5% sodium carbonate (Na_2_CO_3_) aqueous solution in test tubes. The absorbance was measured thrice using a spectrophotometer (V-3000; VWR, Darmstadt, Germany) at 756 nm after 30 min of incubation at room temperature. The results were expressed as mg gallic acid equivalent (GAE) per gram of dried extract [23]. 

Total flavonoid contents were recorded adopting the AlCl_3_ method using quercetin as standard [24]. Briefly, 0.5 mL of test sample was added to 0.5 mL distilled water, 0.15 mL of sodium nitrite (NaNO_2_) solution, 150 µL of AlCl_3_ solution (2%), and 0.2 mL of NaOH (1M). The sample values were recorded at an absorbance of 510 nm using a spectrophotometer after 30 min of incubation at room temperature and results were expressed as mg quercetin equivalents (QE) per gram of dried extract. 

The pH differential method with few alterations was used to determine total anthocyanin contents in the sample [25]. The results were expressed as milligrams of cyanidin 3-glucoside (C3G) per kilogram of dried extract. 

### 2.5. Antioxidant Activity 

*C. carandas* fruit sequential crude extracts were evaluated for DPPH inhibition using the previously described method [26]. Methanol was used to make 1 mM DPPH stock solution, which was kept at −20 °C for analysis. Mixing 10 mL stock and 90 mL methanol yielded 0.1 mM DPPH working solution. To the test tubes, 2 mL DPPH, 2 mL methanol, and 0.2 mL extract (1 g/20 mL) were added. Test tubes were incubated for 30 min in the dark. Via spectrophotometer (UV-Vis 3000, ORI, Hille, Germany), absorbance was measured at 517 nm after incubation. For blank and standard recordings, methanol and quercetin (125 µg/mL) were employed. DPPH % inhibition was recorded using the following equation.

% inhibition = (Control OD − Sample OD/Control OD) × 100

*C. carandas* fruit sequential crude extracts were also tested for ferric-reducing antioxidant potential (FRAP) following the guidelines of Zahin et al. [27]. According to this method, 100 µL of extract (1 g/20 mL) was added to 300 µL of FRAP working solution (containing 300 mmol/L acetate buffer (pH 3.6), 10 mmol/L 2,4,6-tripyridyl-s-triazine (TPTZ) in 40 mmol/L HCl, and 20 mmol/L FeCl_3_ in a ratio of 10:1:1) and then incubated for 30 min at room temperature. Finally, the absorbance was measured at 596 nm with a spectrophotometer and the results were reported as Fe mmol/g of dried extract.

### 2.6. Antimicrobial Activity 

The antimicrobial potential of *C. carandas* sequential crude extracts was tested against UTI-causing bacteria (*Escherichia coli*; ATCC 25922, *Klebsiella pneumoniae*; ATCC 13883), and fungi (*Candida albicans*; BNCC 187382, *Candida glabrata;* BNCC 337348) using the disc diffusion method, as described by Bauer et al. [28]. The bacterial strains were grown on a Nutrient Agar (NA) slant, incubated at 37 °C for 24 h, and kept at 4 °C until further use, whereas the fungal strains were cultured on Sabouraud dextrose agar (SDA) using the spread plate technique. In detail, Mueller-Hinton agar underwent sterilization by autoclaving at a temperature of 121 °C for a period of 15 min. The aseptic method was used to transfer the sterile medium onto pre-sterilized petri dishes, which were then allowed to cool in order to solidify. Bacterial and fungal strains were grown on solid agar using the spread plate method in a sterile environment. Following that, tiny apertures of 6 mm in diameter were generated in the agar and then infused with 30 µL of *C. carandas* dichloromethane, methanol, and 50% methanol extracts at doses of 50 mg/mL, 100 mg/mL, 300 mg/mL, and 400 mg/mL. In contrast, a traditional antibacterial (ciprofloxacin) and antifungal (nystatin) drug were given at a concentration of 5 µg/mL. The petri plates were correctly marked and put in an incubator set at a temperature of 37 °C for a period of 24–48 h for antibacterial activity and 48–72 h for antifungal activity. The activity was recorded as zone of inhibition (ZOI) in mm, wherein a ZOI greater than 8 mm is considered to demonstrate antibacterial and antifungal activity.

### 2.7. Anti-Inflammatory Activity 

#### 2.7.1. In Vitro Assessment 

Membrane stabilization potential of *C. carandas* sequential crude extracts (50–300 µg/mL) was tested per the guidelines of Shinde et al. [29]. Blood samples from healthy humans’ cubital veins were promptly transferred to heparinized tubes. Following centrifugation at 3000 rpm for 5 min, the tubes were rinsed three times with normal saline. The blood volume was then measured in a 10% *v*/*v* suspension using an isotonic buffer solution consisting of 10 mM sodium phosphate buffer at pH 7.4 [30]. After that, 2 mL reaction mixture was made by addition of various crude extracts of *C. carandas* (1 mL) to red blood cell suspension. The method involves two centrifugations, the first performed immediately after mixing the fruit extract and red blood cells suspension, and the second after cooling the mixture. Finally, the supernatant was recovered to check absorbance using a spectrophotometer at 560 nm. The protocols of Mizushima and Kobayashi [31] and Sakat et al. [32] were followed to test the egg albumin and bovine serum albumin denaturation inhibition abilities of sequential crude extracts of *C. carandas* and subsequent fractions. In the egg albumin assay, 0.2 mL egg albumin, phosphate buffer saline, and plant extracts (50, 100, 200, 300 µg/mL) of several concentrations (2 mL) were mixed to produce a reaction mixture of 5 mL. Then, the reaction mixture was incubated, cooled, its absorbance was measured at 660 nm, and the results were expressed as % inhibition. 

Likewise, in the serum albumin assay, 0.05 mL of plant extracts (50, 100, 200, 300 µg/mL) was added to 0.045 mL of serum albumin to produce 0.5 mL of reaction mixture. The reaction mixture was incubated, then a saline phosphate buffer was added, and finally the absorbance was read using a spectrophotometer at 660 nm. 

In all three assays, the positive control was diclofenac sodium and the negative control was phosphate buffer, and the results were outlined as % inhibition. The following equation was used to calculate the % inhibition of plant extracts and standard drug:

% Inhibition = (Abs Control − Abs Treated)/Abs Control × 100

#### 2.7.2. In Vivo Assessment 

In the current study, we tested sequential crude extracts of *C. carandas* for in vivo anti-inflammatory activity using carrageenan- and formaldehyde-induced paw edema models, adhering to the guidelines of Morris (2003) [33]. Eight different groups were treated, each group containing 5 animals, wherein group 1 was the control that received normal saline, group 2 was the positive control that received indomethacin at 100 mg/kg b.w., and the remaining 6 groups were named active groups. Groups 3 and 4 received dichloromethane extract (200 and 400 mg/kg body weight (b.w.)), groups 5 and 6 received methanol extract (200 and 400 mg/kg b.w.), and groups 7 and 8 received 50% methanol extract (200 and 400 mg/kg b.w.). We measured the paw circumference of each rat before the treatment, fed them the aforementioned extracts, and intoxicated the right hind paw’s plantar aponeurosis surface with carrageenan after 30 min. The study used a plethysmometer (UGO-BASILE 7140, Comerio, Italy) to measure the change in paw diameter after 0, 1, 2, and 3 h. The study considered the rise in paw circumference as a sign of swelling.

Brownlee’s [34] guidelines were followed to assess formaldehyde-induced edema inhibition of the *C. carandas* sequential crude extracts. The study divided the albino mice into 8 groups, maintaining the same parameters as the carrageenan model. Before the treatment, the size of each rat’s paw was measured. The mice were then given the extracts, and after 30 min, formaldehyde (100 µL, 4%) was injected into the right hind paw’s plantar aponeurosis. The study used a plethysmometer to measure the change in paw diameter after 0, 3, 6, 12, and 24 h. The study considered the rise in paw circumference as a sign of swelling.

### 2.8. Bioassay-Guided Fractionation 

#### 2.8.1. Liquid-Liquid Partitioning 

*C. carandas* fruit methanol extract exhibiting higher activities was further fractionated using the liquid-liquid partitioning technique using ethyl acetate, dichloromethane, and water. This led to three fractions: fraction A (chloroform), fraction B (ethyl acetate), and fraction C (water). 

#### 2.8.2. Method Optimization for Fractionation 

Fractionation was performed on an HPLC system with a diode array detector (DAD) (1100/1200 series, Agilent, Waldbronn, Germany) with an analytical column (Zorbax-SB-C18, 4.6 × 150 mm, 5 μm, Agilent, Waldbronn, Germany). Five milligrams of ethyl acetate fraction were dissolved in 1 mL of methanol, and after a staying time of 30 min passed through a syringe filter (0.45 µm). The mobile phase was composed of 0.1% trifluoroacetic acid (A), and acetonitrile with 0.1% trifluoracetic acid (B). The sample (10 µL) was injected into the HPLC column. The flow rate was set to 0.5 mL/min and the gradient elution was used: B (15%) in 0–10 min, B (15–30%) in 11–15 min, B (30–60%) in 15–22 min, B (60–90%) in 22–27 min, and B (100%) in 27–30 min. Several detection wavelengths were used including 354, 280, 310, 520 nm to obtain the chromatograms. The process retrieved seven sub-fractions. 

#### 2.8.3. RP-HPLC Sub-Fractionation 

A volume of 70 µL of a sample with a concentration of 1 g/10 mL was loaded onto a Zorbax SB-C18 semi-preparative column (25 × 250 mm, 5 μm particle size, Agilent, Germany). As a consequence, seven sub-fractions, named CCF1-CCF7, were obtained from fraction B (ethyl acetate) of the methanol extract of *C. carandas* fruit. The RP-HPLC sub-fractions showed decreased activity in terms of in vitro antioxidant, anti-inflammatory, and antibacterial properties compared to the parent LLP fraction B and *C. carandas* methanol extract. Subsequently, LLP fraction B underwent additional examination using mass spectrometry. 

### 2.9. LC-ESI-MS/MS Analysis 

Active liquid-liquid partitioned (LLP) fraction or RP-HPLC sub-fractions exhibiting significant biological potential as compared to other fractions and external standards were then analyzed using the LC-MS/MS technique. The detection was performed using direct injection mode with Electron Spray Ionization, in both positive and negative ionization modes. The sample flow rate, temperature of the capillary tube, and mass range were kept to 8 μL/min, 280 °C, and *m*/*z* 50–1000, respectively [35]. Analysis of LC-ESI-MS/MS acquired data was performed using manual Thermo Xcalibur Qual Browser v.3 (Thermo Scientific, Waltham, MA, USA). Structural elucidation was performed using ChemDraw (ChemDraw Ultra 8.0) and then compared with previously published data.

### 2.10. Quantification of Tentatively Identified Compounds Using External Standards

An attempt was made to quantify the phenolic acids identified in the LC-MS/MS analysis of fraction B (*C. carandas* methanol extract). In the present study, chlorogenic acid and ellagic acid were quantified on the basis of standard curves. Sixty milligrams of *C. carandas* fraction B was dissolved in 1 mL methanol (60 mg/mL), and concentration of the phenolic (chlorogenic acid, ellagic acid) and organic acid (quinic acid) standards were 250 µg/mL. One hundred microliters of plant extract and standards was injected into the HPLC system. As stated in Section 2.8.2, all other parameters were identical. UV spectra and retention times of standards were compared to plant extracts for identification and quantification.

### 2.11. Statistical Analysis 

The data from this research are presented as mean ± standard error of means (SEM) for 3 readings. The study compared control and treatment groups using analysis of variance (ANOVA) and Dunnett’s test, with significance set at *p*-value < 0.05 (* *p*-value < 0.05, ** *p*-value < 0.01, *** *p*-value < 0.001). The software employed was Graphpad Prism (Graph Pad Software 8.0.2, San Diego, CA, USA).

## 3. Results

### 3.1. Phytochemical Characterization and Antioxidant Activity 

The successive crude extracts of *C. carandas* were assessed for their total phenolic, flavonoid, and anthocyanin contents, as shown in Table 1. The methanol extract exhibited higher total phenolic contents, whereas the dichloromethane extract showed the lowest levels. The methanol extract had a higher affinity for extracting both total flavonoids and total anthocyanins compared to the 50% methanol and dichloromethane extracts. The quercetin (standard) exhibited the highest level of antioxidant activity in both the DPPH and FRAP experiments, followed by the methanol, 50% methanol, and dichloromethane extracts. Moreover, results are also expressed in terms of quercetin equivalents, determined by comparison to a standard curve generated using known concentrations of quercetin. For the DPPH assay, the *C. carandas* extract at a concentration of 50 mg/mL exhibited a significant % inhibition of 73.0%, which corresponds to a quercetin equivalent concentration of 59.87 µg/mL. Similarly, in the FRAP assay, the 50 mg/mL *C. carandas* extract concentration demonstrated a FRAP value of 51.5 mmol/g, which translates to a quercetin equivalent concentration of 84.53 µg/mL, underscoring its potential as a natural antioxidant source.

### 3.2. Anti-UTI Potential of Sequential Crude Extracts 

*C. carandas* sequential crude extracts were tested for antimicrobial potential against several UTI-causing organisms, and their activity was compared to standard medicines including ciprofloxacin and nystatin (Table 2). The methanol extract showed significant antibacterial activity against *Escherichia coli* (ZOI: 21 ± 0.5 mm) and *Klebsiella pneumoniae* (ZOI: 26 ± 0.5 mm) at 50 µg/mL, surpassing the reference medication, i.e., ciprofloxacin at 5 µg/mL. The methanol extract was effective against *Candida albicans* (ZOI: 12 ± 0.5 mm) and *Candida glabrata* (ZOI: 10 ± 0.5 mm), while nystatin demonstrated strong antifungal activity against both Candida species. 

### 3.3. Anti-Inflammatory Potential of Sequential Crude Extracts 

The current study investigated the anti-inflammatory activity of *C. carandas* sequential crude extracts including in vitro and in vivo models (Table 3). The findings were compared to common anti-inflammatory medicines, namely diclofenac sodium (in vitro) and indomethacin (in vivo). In vitro studies found that the methanol extract significantly inhibited egg albumin denaturation (76 ± 0.13%) and serum albumin denaturation (78 ± 0.5%) at 400 µg/mL. These inhibition rates were similar to the effectiveness of diclofenac sodium, which showed inhibition rates of 91 ± 0.1% and 93 ± 0.12% for egg and serum albumin denaturation, respectively, at 400 µg/mL. The methanol extract significantly inhibited heat-induced hemolysis (48 ± 0.58%) at 400 µg/mL, albeit to a lower degree than diclofenac sodium (86 ± 0.2%). In vivo experiments confirmed the results, with the methanol extract inhibiting carrageenan-induced edema (74%), as well as formaldehyde-induced paw edema (71%) at 400 mg/kg b.w. These inhibition rates are close to those of indomethacin, which inhibited carrageenan-induced edema and formaldehyde-induced paw edema at rates of 79% and 73%, respectively, at 100 mg/kg b.w.

### 3.4. Bioassay-Guided Fractionation

*C. carandas* methanol extract demonstrating notable antioxidant, anti-inflammatory, and antimicrobial properties as compared to other extracts was fractionated adopting the liquid-liquid partitioning (LLP) method as mentioned in Section 2.8. LLP resulted in three different fractions: A (chloroform), B (ethyl acetate), and C (water), and all of these fractions were assessed for the aforementioned biological activities. As can be seen in Table 4, an increase in the biological potential was observed in case of fraction B when compared to the parent methanol extract, but a decline was noticed for fraction A and C. Furthermore, fraction C was then further subjected to RP-HPLC and seven sub-fractions were obtained named CCF1-CCF7. All the sub-fractions were evaluated again, but a decline in biological potential was observed as compared to fraction C and the parent methanol extract. As a result, fraction B was selected for LC-MS/MS for the tentative identification of bioactive metabolites responsible for its biological activities (Table 4).

### 3.5. LC-ESI-MS/MS Analysis and HPLC Quantification

Liquid-liquid partitioned fraction B of *C. carandas* methanol extract demonstrating in vitro biological potential in the aforementioned assays was further tested on LC-ESI-MS/MS for tentative identification of bioactive metabolites (Table 5). The analysis led to the identification of phenolic acids (ellagic acid, quinic acid, chlorogenic acid) and anthocyanins (cyanidin-3-galactoside, peonidin-3-arabinoside, delphinidin-3-galactoside, delphinidin-3-rutinoside). The identification was confirmed using the previous literature which identified quinic acid [36], chlorogenic acid [37], ellagic acid [38], and anthocyanins [39]. Respective to the availability of standards, the present study quantified chlorogenic acid, ellagic acid, and quinic acid at 17.6 µg/mg, 5.9 µg/mg, 3.3 µg/mg, respectively, on a dry weight basis.

## 4. Discussion

The fruit of *C. carandas* underwent sequential extraction using *n*-hexane to remove fatty substances, followed by dichloromethane, methanol, and 50% methanol extractions. This approach was used since the choice of solvent and its polarity may affect the extraction of phytochemicals. *C. carandas* methanol extract was recorded to have higher total phenolic (261 ± 0.8 mg GAE/g), flavonoids (1.2 ± 0.4 mg QE/g), and anthocyanins (112 ± 2.1 mg/kg) contents as compared to 50% methanol and dichloromethane extracts. Previously, *C. carandas* methanol extract was reported to contain notable amounts of phenolic and flavonoid compounds, totaling 15.0 mg GAE/g and 2.92 mg RE/g, respectively [19]. *C. carandas* fruit methanol extract was found to offer greater affinity towards extracting total phenolic (841 mg GAE/100 g) and flavonoid (848 mg CE/100 g) contents as compared to 50% methanol, and 70% methanol extract, which supports the results of the current investigation [40].

The antioxidant activity was evaluated using DPPH and FRAP assays. The DPPH test is a straightforward, precise, and efficient way to check the radical scavenging capacities of plant-based extracts. The approach entails quantifying the shift in DPPH coloration, transitioning from violet to light yellow, as a consequence of the presence of antioxidant compounds [41]. In the present study, antioxidant activity was found consistent with phytochemical potential. Table 1 shows that stable free radical-scavenging inhibition potential varied between 21% and 73%, wherein the *C. carandas* methanol extract was recorded to have higher phytochemical contents and also exhibited the highest inhibition in comparison with the standard quercetin (88% inhibition), while dichloromethane extract showed the lowest results. The findings of Siddiqi et al. [42] and Sarma et al. [43] observed *C. carandas* fruit methanol extract to cause notable inhibition of DPPH at 66% and with an IC_50_ of 27.4 µg/mL, respectively. In another study, *C. carandas* fruit methanol extract scavenged DPPH radicals with an IC_50_ of 46.1 µg/mL as compared to an IC_50_ of 12 µg/mL for standard BHT, which supports the findings of the current investigation. The FRAP (ferric-reducing antioxidant power) test is often used to assess the overall antioxidant abilities of experimental extracts. The FRAP test quantifies the aptitude of an experimental extract to donate electrons by measuring the reduction of ferric ions to ferrous ions [44]. In the present study, FRAP varied between 10.2–51 mmol/g wherein methanol extract displayed more potential as compared to 50% methanol and dichloromethane extracts, and in comparison to the standard quercetin (59.2 mmol/g). *C. carandas* fruit methanol extract was reported to have FRAP inhibition of 90% as compared to 97% inhibition for standard ascorbic acid [16].

The study tested *C. carandas* sequential crude extracts for their antimicrobial potential against UTI-causing organisms (Table 2). Antimicrobial potential was found in order with the phytochemical potential and antioxidant activity. Secondary metabolites, especially flavonoids, can prevent formation of nucleic acid by interacting with enzymes like DNA gyrase and topoisomerase, upset cytoplasmic membrane function, leak intracellular contents, affect energy metabolism pathways like the electron transport chain and ATP synthesis, avert biofilm formation, and modify membrane permeability, causing ion gradients and osmotic pressure imbalances, thereby reducing the likelihood of resistance development [45]. In the present study, *C. carandas* methanol extract exhibited substantial antibacterial activity against *Escherichia coli* and *Klebsiella pneumoniae* with ZOI of 21 mm and 26 mm, respectively, in a dose-dependent manner. These values were even higher than those of the standard ciprofloxacin, which had ZOI of 15 mm and 19 mm, respectively. In addition, *C. carandas* methanol extract also showed strong antifungal activity against *Candida albicans* (ZOI 12 mm) and *Candida glabrata* (ZOI 10 mm) but considerably lower than standard nystatin. This study supports previous findings in the literature that the antimicrobial activity has a direct relation to increasing extract concentration [46]. In contrast, dichloromethane extract had no activity, while 50% methanol extract showed intermediate activity but proved ineffective against fungal strains. The results of the current investigation are in accordance with the previous findings of Agarwal et al. [47] wherein a methanol extract of *C. carandas* leaves demonstrated inhibition of *Escherichia coli* (ZOI 19 mm), i.e., more than standard tetracycline inhibition (ZOI 16.1 mm). *C. carandas* fruit water and methanol extracts were recorded to have significant activity against *Escherichia coli* and *Klebsiella pneumonia* [20,48]. *C. carandas* fruit methanol, ethanol, and water extracts were reported to have significant antibacterial activity against a wide range of pathogenic bacteria including *Pseudomonas aeruginosa*, *Staphylococcus aureus*, *Streptococcus mutans*, *Pseudomonas fluorescens*, and *Salmonella Typhi*, which further strengthens its antibacterial reputation [20,47,48,49]. *C. carandas* leaves methanol extract was found to have significant inhibition against *Candida albicans* with MIC of 0.312 mg/mL as compared to petroleum ether and water extracts wherein no activity was observed [50]. *C. carandas* fruit methanol extract was reported to have slightly higher antifungal activity against *Candida albicans* as compared to dichloromethane extract [51], but considerably lower than the results recorded in the current investigation.

Protein denaturation is a primary source of inflammation, and reliable studies previously described support this connection [52,53]. Anti-inflammatory drugs are known to impart stabilization effects towards human red blood cell membranes against hypotonicity-induced lysis [54]. *C. carandas* fruit methanol extract showed significant inhibition against heat-induced hemolysis, serum albumin denaturation, and egg albumin denaturation inhibition, i.e., 55% (*p* < 0.01), 78% (*p* < 0.001), 76% (*p* < 0.001) at 400 µg/mL, in a dose-dependent manner, whereas standard diclofenac sodium exhibited potent (*p* < 0.0001) inhibition of 86%, 93%, and 91% at the same dose, respectively. A positive correlation was observed between anti-inflammatory activity, phytochemical potential, and antioxidant activity. Similarly, concentration-reliant anti-inflammatory properties of commercial drugs including salicylic acid and phenylbutazone has been previously reported [31]. *C. carandas* methanol extract was reported to show significant anti-inflammatory activity in egg albumin denaturation (58% inhibition), serum albumin denaturation (IC_50_ 188 mg/mL), and heat-induced hemolysis (75% inhibition), i.e., comparable to standard diclofenac sodium [55]. Modifications in the cell surface charges of erythrocyte membranes are believed to be the mechanism behind the anti-inflammatory potential of plant-based extracts and commercial drugs. Antioxidants are known to play an important role in the stabilization of red blood cell membranes by decreasing their chances of collision with other substances that may cause aggregation [56]. Previous studies reported a significant in vitro and in vivo potential of secondary metabolites including phenolic acids, flavonoids, flavonols, and saponins owing to their erythrocyte membrane-stabilizing features and strong affinity for binding cations [30].

In vitro results were then further confirmed in carrageenan- and formaldehyde-induced paw edema animal models. *C. carandas* fruit methanol extract (400 mg/kg b.w.) caused a significant inhibition of carrageenan- and formaldehyde-intoxicated paw edema, at 74% (*p* < 0.0001) and 71% (*p* < 0.0001) when recorded after 3 and 4 h, respectively, comparable to the standard indomethacin (100 mg/kg b.w.) inhibition of 79% and 73%, respectively (*p* < 0.0001). The findings are in accordance with Anupama and Madhumitha [57], wherein *C. carandas* fruit methanol extract at 400 mg/kg reduced carrageenan-induced inflammation up to 76% after 2 h in rats as compared to 80% inhibition of standard Indomethacin. Recently, *C. carandas* ripe fruit methanol extract demonstrated notable inhibition against carrageenan-induced edema (66%) as compared to standard inhibition of 78% at the end of 3 h in rats due to the presence of flavonoids, phenolic acids, and anthocyanins [58]. In contrast, the present study observed dichloromethane extract to have non-significant effects, and 50% methanol extract to have moderate anti-inflammatory potential.

Activity-guided fractionation is an effective and successful technique in identification, isolation, and purification of biologically active metabolites [59,60]. In the present study, liquid-liquid partitioned fraction B (chloroform) exhibited more antioxidant and anti-inflammatory activity while no difference was observed in antimicrobial potential as compared to the parent methanol extract. RP-HPLC sub-fractionation of fraction B further yielded seven fractions, but the activity was declined. As a consequence, fraction B was subjected to LC-MS analysis and tentatively identified to have phenolic acids and anthocyanins, with the activity attributed to the presence of these compounds. Reliable literature has been cited to show the biological potential of compounds tentatively identified in the present study. Chlorogenic acid was reported to inhibit carrageenan-induced oxidative stress and inflammatory reactions by inhibiting pro-inflammatory mediators [61]. Ellagic acid was reported to provide protection against carrageenan-induced acute inflammation by inhibiting nuclear factor kappa B, inducible cyclooxygenase, and proinflammatory cytokines. It also enhanced interleukin-10 via an antioxidant mechanism [62]. Likewise, antibacterial activities of chlorogenic acid, ellagic acid, and quinic acid have been reported previously [63,64,65] mediated by membrane disruption properties. Anthocyanins-rich extracts of berry fruits were also found to have significant antioxidant, anti-inflammatory, and antimicrobial properties [66,67,68]. It can be concluded that biological properties of *C. carandas* fruit methanol extract (fraction B) is due to the synergistic effect of the aforementioned phenolic acids and anthocyanins.

## 5. Conclusions

An in-depth analysis of *C. carandas* fruit revealed its phytochemical makeup, which includes phenolic acids and anthocyanins. Furthermore, it exhibited promising biological potential such as antioxidant, anti-inflammatory, and antibacterial effects, thereby validating its ancient use in therapeutic procedures. *C. carandas*’ antioxidant characteristics make it a desirable option for utilization in cosmetics and as a natural preservative for fats, oils, and the meat industry as a green-label alternative to synthetic antioxidants like BHT and BHA. In addition, the use of anthocyanins, which are renowned for their vibrant hues, presents promising opportunities as organic pigments in the food and cosmetics sectors. Robust anti-inflammatory activity, both in vitro and in vivo, suggests their utility in managing inflammatory conditions. Additionally, their antibacterial efficacy against common urinary tract infection pathogens underscores their potential as therapeutic agents in combating microbial infections and also as natural preservatives. Further exploration into the integration of these extracts into functional foods or nutraceuticals could pave the way for innovative solutions in both sectors, addressing the growing demand for natural and effective health-promoting ingredients. Future studies should also give priority to the isolation of specific bioactive compounds, the clarification of their mechanisms of action, and the exploration of their potential in the development of pharmaceuticals, cosmetics, and food products.

## Figures and Tables

**Table 1:** Phytochemical contents of *C. carandas* sequential crude extracts.

Phytochemicals/Antioxidant Activity	DCM	MeOH	50% MeOH	Quercetin
TPC (mg GAE/g)	180 ± 0.134 ^a^	261 ± 0.813 ^c^	211 ± 0.163 ^b^	--
TFC (mg QE/g)	0.32 ± 0.731 ^b^	1.22 ± 0.421 ^c^	0.21 ± 0.512 ^a^	--
TAC (mg/kg)	0.91 ± 0.612 ^a^	112 ± 2.12 ^c^	76.0 ± 0.582 ^b^	--
DPPH (% inhibition)	21.0 ± 0.522 ^a^	73.0 ± 0.403 ^c^	59.0 ± 0.122 ^b^	88.0± 0.112 ^d^
FRAP (mmol/g)	10.2 ± 0.214 ^a^	51.5 ± 1.51 ^c^	43.3 ± 1.11 ^b^	59.2± 0.121 ^d^

All values are mean of triplicate determinations. Different lower case letters within a column and different numbers within a row are significantly different at *p* < 0.05. TPC, total phenolic contents. TFC, total flavonoids contents. TAC, total anthocyanins contents. DPPH, 2,2-diphenyl-1-picrylhydrazyl. FRAP, ferric-reducing antioxidant power. DCM, dichloromethane. MeOH, methanol.

**Table 2:** Anti-urinary tract infection potential of *C. carandas* sequential crude extracts.

Microbe Type	Type of Extracts			Standard Drugs	
	DCM	MeOH	50% MeOH	Ciprofloxacin	Nystatin
	ZOI at 50 µg/mL (mm)			ZOI at 5 μg/mL (mm)	100 Units/mL (mm)
*Escherichia coli* (Gram-negative)	NA	21.0 ± 0.231 ^c^	11.0 ± 0.126 ^a^	15.0 ± 0.251 ^b^	--
*Klebsiella pneumoniae* (Gram-negative)	NA	26.0 ± 0.112 ^c^	9.0 ± 0.117 ^a^	19.0 ± 0.512 ^b^	--
*Candida albicans*	NA	12.0 ± 0.543 ^b^	4.0 ± 0.237 ^a^	--	21.0 ± 0.548 ^c^
*Candida glabrata*	NA	10.0 ± 1.38 ^a^	NA	--	16.0 ± 0.547 ^b^

All values are mean of triplicate determinations. Different lower case letters within a column and different numbers within a row are significantly different at *p* < 0.05. DCM, dichloromethane. MeOH, methanol. 50% MeOH, 50:50 water and methanol. ZOI, zone of inhibition. DCM, dichloromethane. MeOH, methanol. ZOI, zone of inhibition. NA, no activity.

**Table 3:** Anti-inflammatory potential of *C. carandas* sequential crude extracts.

In Vitro Assays	Type of Extract			Standard	
	DCM	MeOH	50% MeOH	Diclofenac Sodium (In Vitro)	Indomethacin (In Vivo)
	% Inhibition at 400 µg/mL			% Inhibition at 400 µg/mL	% Inhibition at 100 mg/kg b.w.
Egg albumin denaturation	11.0 ± 0.01 ^ns^	76.0 ± 0.131 ***	51.0 ± 0.932 **	91.0 ± 0.126 ****	--
Serum albumin denaturation	14.0 ± 0.02 ^ns^	78.0 ± 0.546 ***	54.0 ± 0.351 **	93.0 ± 0.124 ****	--
Heat-induced hemolysis	8.0 ± 0.631 ^ns^	55.0 ± 0.581 **	43.0 ± 0.951 **	86.0 ± 0.232 ****	--
*In vivo study*	% inhibition at 400 mg/kg b.w.				
Carrageenan-induced paw edema	21.0 *	74.0 ****	39.0 **	--	79.0 ****
Formaldehyde-induced paw edema	14.0 ^ns^	71.0 ****	32.0 **	--	73.0 ****

All values are mean of triplicate determinations. Different lower case letters within a column and different numbers within a row are significantly different at *p* < 0.05 (* *p* < 0.05, ** *p* < 0.01, *** *p* < 0.001, **** *p* < 0.0001). ns, non-significant. MeOH, methanol. % inhibition = inhibition as compared to control.

**Table 4:** Biological activities of bioassay-guided liquid-liquid partitioned fractions and RP-HPLC sub-fractions.

Assay/Activity	LLP Fraction of Methanol Extract	RP-HPLC Sub-Fractions of LLP Fraction B		Standards			
	B (50 mg/mL)	CCF4 (50 mg/mL)	CCF7 (50 mg/mL)	Qr (125 µg/mL)	Dfs (400 µg/mL)	Cip (5 μg/mL)	Nys (100 units/mL)
DPPH (% inhibition)	76.1 ± 0.211 ^c^	65.5 ± 0.821 ^b^	41.4 ± 1.10 ^a^	88.4 ± 0.541 ^d^	--	--	--
FRAP (mmol/g)	59.2 ± 1.10 ^c^	43.2 ± 0.551 ^b^	34.2 ± 1.52 ^a^	59.4 ± 0.223 ^c^	--	--	--
Anti-inflammtory activity (% inhibition at 400 µg/mL)							
Egg albumin denaturation	82.4 ± 0.541 ***	69.4 ± 0.359 **	57.2 ± 0.312 **	--	91.2 ± 0.124 ****	--	--
Serum albumin denaturation	85.1 ± 1.81 ***	52.1 ± 0.431 **	48.4 ± 0.223 **	--	93.3 ± 0.115 ****	--	--
Heat-induced hemolysis	66.2 ± 1.52 **	29.4 ± 0.611 *	37.1 ± 0.212 *	--	86.1 ± 0.241 ****	--	--
Antibacterial activity (ZOI mm) 50 µg/mL							
*Escherichia coli*	18.4 ± 1.12 ^d^	8.1 ± 0.148 ^b^	6.1 ± 0.259 ^a^	--	--	15.4 ± 0.15 mm ^c^	--
*Klebsiella pneumoniae*	22.2 ± 1.221 ^d^	7.4 ± 0.124 ^b^	4.2 ± 0.521 ^a^	--	--	19.1 ± 0.545 mm ^c^	--
*Candida albicans*	11.4 ± 1.51 ^a^	NA	NA	--	--	--	21.4 ± 0.541 mm ^b^
*Candida glabrata*	9.1 ± 0.841 ^a^	NA	NA	--	--	--	16.1 ± 0.543 mm ^b^

All values are mean of triplicate determinations. Different lower case letters within a column and different numbers within a row are significantly different at *p* < 0.05 (* *p* < 0.05, ** *p* < 0.01, *** *p* < 0.001, **** *p* < 0.0001). % inhibition = inhibition as compared to control. Qr, quercetin. Dfs, diclofenac sodium. Cip, ciprofloxacin. Nys, nystatin. LLP, liquid-liquid partitioned fraction. CCF, *Carissa carandas* sub-fraction.

**Table 5:** LC-ESI-MS/MS analysis of bioactive metabolites from active liquid-liquid partitioned fraction B of *C. carandas* methanol extract.

Tenetaive Compound	Detection Mode	Molecular Weight *m*/*z*	Fragments	Reference
Quinic acid	Negative	191	191, 173, 127.1, 85.3	[36]
Chlorogenic acid	Negative	353	191, 173.1	[37]
Ellagic acid	Negative	301	301, 285, 283.1, 256.9, 229, 178.9	[38]
Cyanidin-3-galactoside	Negative	449	286.9	[39]
Peonidin-3-arabinoside	Positive	433	283	[39]
Delphinidin-3-galactoside	Positive	465	303	[39]
Delphinidin-3-rutinoside	Positive	611	302.9	[39]

## Data Availability

All the data are presented in the manuscript.
